# Complete genome sequences of KPC-producing *Klebsiella pneumoniae* isolates pre- and post-development of meropenem/vaborbactam resistance

**DOI:** 10.1128/mra.01447-25

**Published:** 2026-03-10

**Authors:** Egon A. Ozer, Sophie H. Nozick, Nidhi Singh, Jackson V. Watkins, Alan R. Hauser, Zackery P. Bulman

**Affiliations:** 1 Division of Infectious Diseases, Department of Medicine, Northwestern University Medical School 576055, Chicago, Illinois, USA; 2 Department of Microbiology-Immunology, Northwestern University Feinberg School of Medicine 12244, Chicago, Illinois, USA; 3 Department of Pharmacy Practice, Retzky College of Pharmacy, University of Illinois Chicago 14681, Chicago, Illinois, USA; University of Pittsburgh School of Medicine, Pittsburgh, Pennsylvania, USA

**Keywords:** *Klebsiella pneumoniae*, meropenem/vaborbactam, KPC, antibiotic resistance, beta-lactamases

## Abstract

*Klebsiella pneumoniae* carbapenemase-producing *K. pneumoniae* (KPC-Kp) are high-priority pathogens. Although meropenem/vaborbactam is a preferred treatment, resistance can emerge. Two KPC-Kp isolates from the Centers for Disease Control and Prevention(CDC) Antimicrobial Resistance Isolate Bank were sequenced before and after development of meropenem/vaborbactam resistance following treatment with this antibiotic in the hollow fiber infection model (HFIM).

## ANNOUNCEMENT

The *Klebsiella pneumoniae* carbapenemase (KPC) enzyme is a common carbapenemase encountered in carbapenem-resistant *K. pneumoniae* isolates from patients in the United States. One of the preferred antibiotics for treatment of KPC-Kp is meropenem/vaborbactam, which employs a boronic acid-based β-lactamase inhibitor, vaborbactam, to neutralize the hydrolyzing capabilities of KPC. Resistance to meropenem/vaborbactam has been detected in KPC-Kp isolates *in vitro* (1) and in patients (2) and is primarily caused by loss-of-function mutations in the outer membrane porins (e.g., *ompK36*) and/or increased *bla*_KPC_ copy number. Herein, we provide the complete chromosome and plasmid sequences for: (i) KPC-Kp isolates AR-1049 and AR-1054 from the CDC Antimicrobial Resistance Isolate Bank, (ii) AR-1049 and AR-1054 grown in the HFIM for 168 h without antibiotic (identified by suffix “-GC”), and (iii) mutants of these isolates that each became resistant to meropenem/vaborbactam (MICs >32/8 mg/L) following antibiotic treatment for 168 h in the HFIM (identified by suffix “-MV”). Bacterial growth over 168 h in the HFIM was performed at 37°C in cation-adjusted Mueller-Hinton broth (CAMHB). Fresh CAMHB was continuously infused into the system throughout the experiment to maintain nutrients and remove waste. Experiments used to generate the resistant mutants are described in greater detail in our companion article (3).

For short-read sequencing of bacteria grown in the HFIM, isolates were initially sub-cultured on MHA, and individual colonies were transferred to Mueller-Hinton broth for DNA extraction. A commercial kit (ZymoBIOMICS Quick-DNA MiniPrep Kit) was used to extract DNA from the samples in accordance with the manufacturer’s protocol. The final purified DNA was resuspended in 60 µL of the manufacturer-supplied DNA elution buffer. To generate paired-end reads of 150 bp, libraries were prepared using the Illumina DNA Prep kit (Illumina, Inc.) and then sequenced on an Illumina NovaSeq X.

For long-read sequencing, a single colony was picked and was grown overnight in LB at 37°C in a shaking incubator. The following day, DNA was extracted using a DNA Tissue Kit on the Qiagen EZ2 Connect. Libraries were prepared from unsheared genomic DNA with the Oxford Nanopore Native Barcoding Kit 24 without size selection. Long-read sequencing was performed on a GridION sequencer using R10.4.1 flow cells run for 72 h, and basecalling was performed using Dorado v7.6.7 using the super-accurate model (v4.3.0, 400 bp) with a minimum quality score of 10 (https://github.com/nanoporetech/dorado).

Complete genome sequences were assembled using Autocycler v0.2.1 with default parameters (4). Briefly, for each genome, eight subsamples of the long read sets produced by Autocycler were assembled using Flye v2.9.5, Raven v1.8.3, and miniasm v0.3, each using default parameters, to generate 24 assemblies (5–8). Assemblies were reconciled into circular consensus assemblies using Autocycler. Circular assembly sequences were reoriented using dnaapler v0.8.1 to begin at the *dnaA* gene (chromosome) or *repA* gene (plasmids) and polished first with Nanopore reads using medaka v2.0.1 and then with corresponding Illumina reads using polypolish v0.6.0 and pypolca v0.3.1 (9, 10). Annotation was performed using the NCBI Prokaryotic Genome Annotation Pipeline (PGAP) v6.10. Species were identified by nucleotide BLAST of the assembly 16S rRNA sequence against the NCBI 16S ribosomal RNA database. Assembly statistics and accession numbers are provided in Table 1.

**TABLE 1:** Assembly statistics and accession numbers for two parental strains (AR-1049 and AR-1054) as well as the mutants that were grown for 168 h without antibiotic (identified by suffix “-GC”) or following exposure to meropenem/vaborbactam (identified by suffix “-MV”) in the hollow fiber infection model

				Average coverage^*a*^				SRA accession		
Strain	Sequence	Bases	%GC	Nanopore	Illumina	Nanopore N50	BioSample	Nanopore	Illumina	Assembly
AR-1049	**Total genome**	**5,765,901**	**57.01**	363.1	73.0	15917	SAMN53681260	SRR36316120	SRR13338365	JBUBNU010000000
	Chromosome	5,305,401	57.5	340.7	64.8					
	Plasmid 1	214,836	52.8	470.0	83.6					
	Plasmid 2	125,021	53.6	748.1	152.4					
	Plasmid 3	79,489	45.6	507.6	159.4					
	Plasmid 4	41,154	53.5	298.0	108.4					
AR-1049-GC	**Total genome**	**5,766,570**	**57.01**	206.9	65.9	16987	SAMN53681261	SRR36316119	SRR36316114	JBUBNT010000000
	Chromosome	5,306,070	57.5	194.8	46.4					
	Plasmid 1	214,836	52.8	261.5	107.4					
	Plasmid 2	125,021	53.6	411.9	210.6					
	Plasmid 3	79,489	45.6	301.0	310.3					
	Plasmid 4	41,154	53.5	168.5	142.4					
AR-1049-MV	**Total genome**	**5,721,708**	**57.01**	100.5	75.0	25641	SAMN53681262	SRR36316118	SRR36316113	JBUBNS010000000
	Chromosome	5,303,191	57.5	92.7	51.5					
	Plasmid 1	216,524	52.8	143.0	107.7					
	Plasmid 2	125,021	53.6	219.7	194.5					
	Plasmid 3	79,489	45.6	173.2	326.2					
	Plasmid 4	41,154	53.5	76.1	152.1					
AR-1054	**Total genome**	**5,817,720**	**57.03**	227.3	97.0	25324	SAMN53681263	SRR36316117	SRR13338360	JBUBNR010000000
	Chromosome	5,439,030	57.3	219.7	92.8					
	Plasmid 1	208,035	52.9	325.1	100.7					
	Plasmid 2	113,639	53.9	362.7	124.5					
	Plasmid 3	43,380	46.8	137.7	104.5					
	Plasmid 4	13,636	53.7	160.7	255.0					
AR-1054-GC	**Total genome**	**5,817,720**	**57.05**	192.1	71.4	25474	SAMN53681264	SRR36316116	SRR36316112	JBUBNQ010000000
	Chromosome	5,439,030	57.3	186.4	58.2					
	Plasmid 1	208,035	52.9	268.3	95.3					
	Plasmid 2	113,639	53.9	281.2	148.4					
	Plasmid 3	43,380	46.8	126.0	150.5					
	Plasmid 4	13,636	53.7	154.6	190.9					
AR-1054-MV	**Total genome**	**5,721,708**	**57.06**	513.0	49.7	15,297	SAMN53681265	SRR36316115	SRR36316111	JBUBNP010000000
	Chromosome	5,441,430	57.3	430.0	39.3					
	Plasmid 1	273,529	53.3	1957.8	135.0					
	Plasmid 2	43,380	46.8	509.0	85.5					
	Plasmid 3	13636	53.7	811.569	170.773					

^*a*^ Total coverages are total read bases divided by assembly size. Chromosome and plasmid read coverages are average depths based on read alignments against the assemblies.

## Data Availability

Sequence data are deposited under NCBI BioProject accession PRJNA1374223.
